# Mitochondria-Derived Damage-Associated Molecular Patterns in Sepsis: From Bench to Bedside

**DOI:** 10.1155/2019/6914849

**Published:** 2019-05-08

**Authors:** Sicheng Li, Qiongyuan Hu, Jinjian Huang, Xiuwen Wu, Jianan Ren

**Affiliations:** ^1^Research Institute of General Surgery, Jinling Hospital, Nanjing Medical University, Nanjing 210002, China; ^2^Lab for Trauma and Surgical Infections, China; ^3^Medical School of Nanjing University, Nanjing 210093, China; ^4^School of Medicine, Southeast University, Nanjing 210009, China

## Abstract

Sepsis is one of the most serious health hazards. Current research suggests that the pathogenesis of sepsis is mediated by both pathogen-associated molecular patterns (PAMPs) and damage-associated molecular patterns (DAMPs). Mitochondria are among the most important organelles in cells and determine their life and death. A variety of mitochondria-derived DAMPs (mtDAMPs) are similar to bacteria because mitochondria are derived from bacteria according to the mitochondrial endosymbiotic theory. Their activated signaling pathways extensively affect organ functions, the immune system, and metabolic functions in sepsis. In this review, we describe the essential roles of mtDAMPs in sepsis and discuss their research prospects and clinical importance.

## 1. Introduction

In recent years, although the mortality rate of patients with sepsis has decreased significantly due to the improved technology of life support, the problem of long-term stay in the intensive care unit (ICU) remains and is on the rise [1]. In 2016, Sepsis 3.0 proposed that sepsis is a life-threatening organ dysfunction caused by a disordered response to infections [2]. The definition of sepsis has developed from a simple inflammatory response to the comprehensive responses of the body to infections, which reveals the essence of sepsis and has an importance for future research and treatment. With the improved understanding of pathogenesis, the inflammatory response of pathogen-associated molecular patterns (PAMPs) in the body cannot fully explain the abnormal physiological alterations and multiorgan dysfunctions in sepsis. Therefore, damage-associated molecular patterns (DAMPs) have received much attention in the field of sepsis for the past few years [3]. DAMPs can bind to their associated pattern recognition receptors (PRRs) and activate a series of inflammatory pathways that account for the occurrence, development, and outcome of diseases [4].

Mitochondrial dysfunction seriously affects cell energy metabolism and releases a large number of components into the cytosol or extracellular space, resulting in a series of biochemical reactions, such as apoptosis, necrosis, and inflammation. According to the endosymbiotic theory of mitochondria, mitochondria may have originated from energy-producing bacteria [5]. Thus, mitochondria-derived DAMPs (mtDAMPs) and PAMPs have many similarities [6].

**Figure 1:**
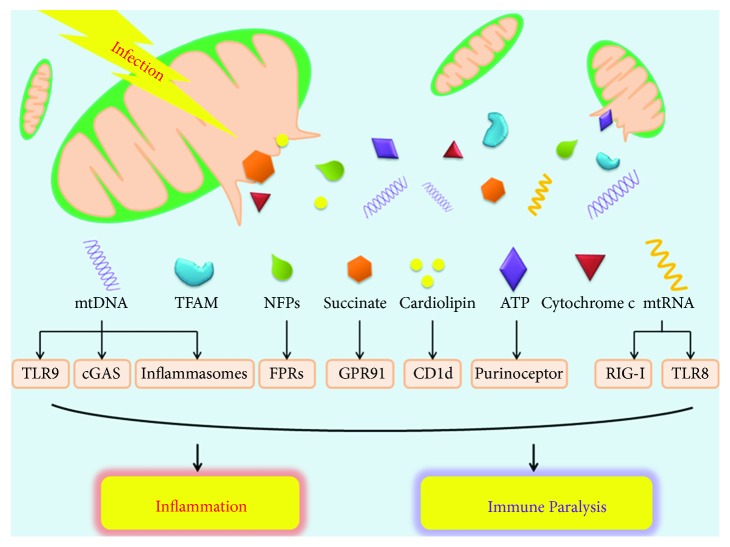
Overview of mtDAMPs in sepsis.

The complex immune responses triggered by mtDAMPs, including cytokine storms and immunosuppression, mediate the pathophysiological process of sepsis (Figure 1). Our recent findings demonstrate that plasma mtDAMP levels are associated with sepsis, multiorgan dysfunction syndrome (MODS), and death in patients with intra-abdominal infection caused by severe abdominal trauma. The level of mtDAMPs in urine can identify acute kidney injury [7] and provide the basis for initiation of renal replacement therapy [8]. We have confirmed that escaped mtDNA [9] and ATP [10] deteriorate the intestinal hemostasis in sepsis, and destruction of the intestinal barrier probably initiates the MODS. Here, we review the role of mtDAMPs in the pathogenesis of sepsis and discuss their prospects for future research on sepsis and their clinical application.

## 2. mtDNA

In all types of sepsis-related mtDAMPs, mtDNA has been widely investigated, but our understanding of it is still incomplete [11]. Here, we discuss the role of mtDNA in a more in-depth way, not limited to the purely inflammatory injury effect.

Many clinical studies have shown that mtDNA is important for the prognosis of sepsis [12–14]. Our team has demonstrated that among patients with abdominal infections caused by severe abdominal trauma, plasma mtDNA content in those who have died is significantly higher than those of survivors [8]. We have also demonstrated in animal cecal ligation and puncture (CLP) models that elevated mtDNA levels in urine are associated with mitochondrial dysfunction and renal injury [7]. mtDNA in urine of critically ill patients is negatively related to the glomerular filtration rate, suggesting that mtDNA in urine is an effective biomarker for predicting acute kidney injury and hospitalization mortality in sepsis and provides a basis for initiation of continuous renal replacement therapy [15]. Bhagirath et al. [16] demonstrated that mtDNA in plasma of sepsis patients was 50 times higher than that in a healthy control group. In addition, high concentration of mtDNA can promote amplification of inflammatory response by delaying apoptosis of neutrophils. Lubkin et al. [17] assessed studies published between 1971 and 2017 that measured extracellular mtDNA in acutely ill patients. They concluded that elevated mtDNA has clinical value for risk prediction and clinical decision support systems in critically ill patients.

Picca et al. [18] have suggested that mtDNA promotes skeletal muscle catabolism through cGAS- (cyclic guanosine monophosphate-adenosine monophosphate synthase-) STING (stimulator of interferon genes), Toll-like receptor (TLR) 9, NOD-like receptor family, pyrin domain-containing protein 3 (NLRP3), and other pathways. Inflammatory responses mediated by interleukin- (IL-) 6 and tumor necrosis factor (TNF) have long been considered important mechanisms of skeletal muscle decomposition, and mtDNA can induce the production of relevant inflammatory factors through multiple signaling pathways [19]. mtDNA may be one of the causes of muscular atrophy and weakness in sepsis [20].

The elimination of mtDNA may be an effective means to improve the immune function of patients with sepsis. Martinez-Quinones et al. [21] observed in the pilot study that abdominal cavity irrigation could reduce the concentration of ND6 (a kind of mtDAMPs) in peritoneal fluid of open abdomen patients. However, peritoneal lavage does not reduce the level of mtDNA in the abdominal cavity. They have speculated that this might be related to degradation of mtDNA by DNase in the abdominal cavity. However, that research had the drawbacks of too small sample size (*n* = 10) and a low level of evidence (level of evidence: prospective study, case series, level V). mtDNA is closely related to the prognosis of critically ill patients, especially sepsis patients, and may be an effective biological marker to determine the prognosis of critically ill patients. Removing mtDNA may be an effective means to protect important organs in sepsis.

### 2.1. TLR9

TLR9 is the most classical recognition receptor of mtDNA [22, 23]. Previous studies have shown that mtDNA causes serious damage to the lungs [23] and kidneys [24] via TLR9 in sepsis. Recent studies have updated our understanding of TLR9. Hotz et al. [25] demonstrated that red blood cells can bind to mtDNA homeostatically through TLR9 to scavenge it, thereby alleviating pulmonary inflammation in sepsis. This is contrary to the traditional view that TLR9 is an important injury factor in sepsis. Therefore, in the study of sepsis, we should also focus on the dual roles of this receptor. Traditionally, the pathophysiological mechanism of sepsis has been considered to be excessive systemic inflammatory responses followed by immunosuppression. However, recent studies have suggested that inflammation and immunosuppression may occur simultaneously, and immunosuppression is not an overcompensation for inflammation [26]. mtDAMPs may be the key to both inflammation and immunosuppression [27]. The spleen (an important immune organ) in patients with sepsis is severely damaged, and its tissue structure is destroyed. CD8^+^ dendritic cells in the spleen are significantly reduced, and the expression level of soluble programmed death ligand 1, an immunosuppressive factor, is significantly increased [28]. When TLR9 is knocked out, the above immune function damage is significantly alleviated [29], while activation of TLR9 is associated with apoptosis of spleen cells [11]. Immune paralysis in sepsis may be triggered by activation of TLR9 by mtDNA.

### 2.2. cGAS-STING

mtDNA-recognized cGAS-STING has attracted extensive attention since it came into researchers' sight and has become a star pathway in the field of immunity [30–32]. We believe that interferon- (IFN-) I response induced by the cGAS-STING pathway can lead to increased inflammation, apoptosis, necroptosis, and pyroptosis [33]. Damage to autophagy, which has been shown to protect against multiple organ damage in animal models of sepsis, may result in aberrant activation of STING signaling, leading to uncontrolled inflammation and cell death [33]. Discovery of the cGAS-STING signaling pathway opens up a new route for the study of the sepsis mechanism and further emphasizes the crucial role of mtDNA in sepsis.

The chemical nature of cGAS, which can produce endogenous CDN: 2′-3′cyclic AMP-GMP (cGAMP), is nucleic acid transferase. cGAMP induces the STING expression, and STING then recruits tank-binding kinase (TBK) 1 to activate IFN regulatory factor (IRF) 3 and induce the expression of IFN-I and other IFN-stimulated genes. In the presence of I*κ*B kinase (IKK), STING phosphorylates I*κ*B and releases it from nuclear factor- (NF-) *κ*B. NF-*κ*B is activated from an inhibitory state and is translocated into the nucleus to generate inflammatory factors such as TNF-*α* and IL-1*β* [34]. Zhou et al. [35] have demonstrated that levels of cGAMP synthesis are related to stimulation of its DNA structure. Enhanced DNA-length specificity restrains human cGAS activation.

Activation of cGAS-STING is believed to strengthen the antitumor immune response and inhibit tumor growth [36, 37]. However, the role of cGAS-STING in sepsis is controversial. Zeng et al. [38] have proposed anaplastic lymphoma kinase (ALK) as a new therapeutic target for sepsis. ALK, which is abundantly expressed in human and murine monocytes and macrophages, directly interacts with epidermal growth factor receptor to trigger serine-threonine protein kinase AKT phosphorylation and activate IRF3 and NF-*κ*B signaling pathways, enabling STING-dependent rigorous inflammatory responses. However, Blasco et al. [39] have argued that no detectable expression of the ALK receptor is found in mouse and human monocytes and macrophages; thus, replication studies are needed. In this part, cGAS-STING regulates the immune responses to mtDNA, and its resulting effect in sepsis remains further investigated.

### 2.3. NLRP3 Inflammasome

NLRP3 inflammasome, consisting of NLRP3, apoptosis-associated speck-like protein (ASC), and caspase-1, is a member of the NOD-like receptor (NLR) family [40]. Excessive mitochondrial reactive oxygen species (ROS) lead to changes in mitochondrial membrane potential and permeability, which lead to decoupling of the mitochondrial electron transport chain to produce more ROS in turn [40–42]. Mitochondrial damage promotes the release of mtDNA into the cytoplasm, consequently activating NLRP3 inflammasome and upregulating IL-1*β* and IL-18 inflammatory factors by caspase-1 [43, 44]. Although the NLRP3 inflammasome is believed to play a central role in numerous inflammatory, immune, and metabolic diseases, the mechanism that controls its activation is poorly understood. Recent studies have further revealed the activation process of NLRP3. Zhong et al. [45] have confirmed that new mtDNA synthesis is a key step in NLRP3 activation. Expression of CMPK2, a rate-limiting enzyme for *de novo* mtDNA synthesis, is induced by TLR4/myeloid differentiation primary response 88 (MyD88)/IRF1 or TLR4/TIR-domain-containing adapter-inducing interferon-*β* (TRIF)/IRF1 signaling. CMPK2-dependent mtDNA synthesis is necessary for the production of oxidized mtDNA fragments. Oxidative mtDNA can directly bind to NLRP3 to activate the multiprotein complexes [46]. MtDNA may play a positive feedback regulatory role in the activation of the NLRP3 inflammasome. NLRP3 is recruited to the dispersed trans-Golgi network, which serves as a scaffold for NLRP3 aggregation into multiple puncta, leading to polymerization of the adaptor protein ASC [47]. Volt et al. [48] have demonstrated that activation of the NLRP3 inflammasome increases susceptibility to sepsis in aged mice. Melatonin administration can relieve mitochondrial damage and inflammation in sepsis and enhance mitochondrial function in nonseptic aged mice. The treatment of elderly sepsis patients has always been one of the difficult problems for ICU physicians, and Volt's work provides a new approach for this problem.

## 3. mtRNA

Like nuclear DNA, mtDNA can be transcribed into mtRNA. Dhir et al. [49] have shown that there is a highly unstable native mitochondrial double-stranded RNA (dsRNA) species at a single-cell level, which can be degraded by polynucleotide phosphorylase, such as PNPase. PNPase is located in the mitochondrial intermembrane space and has the dual regulatory effect of preventing the formation and release of mitochondrial dsRNA. When PNPase is deficient, mitochondrial dsRNA is produced in large quantities and accumulates in the cytosol. Retinoic acid-inducible gene- (RIG-) I detects dsRNA and activates the transcription factors NF-*κ*B and IRF3 through the mitochondrial protein mitochondrial antiviral signaling protein (MAVS) [50]. Kruger et al. [51] have demonstrated that mitochondrial dsRNA can also be identified by TLR8, relying on the MyD88 signal transduction pathway. TLR8 can also participate in the regulation of cell proliferation, differentiation, and apoptosis via the mitogen-activated protein kinase (MAPK) signaling pathway [52]. Synergistic effects exist between multiple receptors, and different mtDAMPs are released simultaneously, leading to stronger signal pathway activation. RIG-I and STING recognize RNA and DNA, respectively, but the downstream of their signaling pathways is correlated. Cheng et al. [53] have demonstrated that activation of this RNA-sensing pathway requires prior STING activation and works synergistically with the DNA-sensing pathway to induce an immune response during *Mycobacterium tuberculosis* infections. Nazmi et al. [54] have proved that RIG-I binds to Japanese encephalitis virus RNA and then synergizes with STING to induce expression of IFN-I, thus leading to antiviral activity. Knockdown of STING decreased the expressions of various inflammatory signaling molecules and increased the intracellular viral load. This also partly explains the synergistic effect between cGAS and TLR4 observed in the past [55]. IFN-I has been approved for treatment of a variety of virus-related diseases since the last century, but its role in sepsis has been controversial. IFN-I inhibits production of pro-IL-1*β* and cleavage of pro-IL-1*β* into mature IL-1*β*, ultimately inhibiting secretion of IL-1*β* by blocking the activity of inflammasomes [56]. In addition, the increase of IFN-I downregulates expression of IFN-*γ* receptor IFNGR1 in the form of negative feedback, thus inhibiting expression of IFN-*γ*-induced MHC II molecules [57]. However, Dejager et al. [58] have shown that in sepsis, IFN-I inhibits accumulation of neutrophils to inflammatory sites by inhibiting the chemokine KC, thereby increasing the risk of sepsis mortality. Inhibition of IFN-I signaling pathways such as RIG-I/MAVS/IFN-I and cGAS/STING/IFN-I may become new effective therapeutic targets for sepsis. This also proves that mtDAMPs work synergetically, and the release of multiple mtDAMPs may cause greater damage.

## 4. Cytochrome c

Cytochrome c, a small hemeprotein, is an essential component of the electron transport chain. Cytochrome c is located in the gap between the inner and outer mitochondrial membranes and binds to the inner membrane [59]. In the cytoplasm, cytochrome c is involved in the formation of apoptosomes which induce cell apoptosis. If cytochrome c migrates from the cytoplasm into the extracellular space or circulation, it may trigger inflammatory reactions. However, no clear PRR of cytochrome c has been identified, which is the focus of future research on cytochrome c. Sepsis often develops into MODS [60]. Clinical studies have shown that the level of cytochrome c in plasma or urine can be used as a biomarker for heart [61], liver [62], kidney [63], and other important organ damages. Andersen et al. [64] showed that the level of cytochrome c in plasma of patients with septic shock is significantly higher than that in healthy controls. They also observed a positive correlation between the level of cytochrome c upon admission and the level of lactic acid, which is a well-acknowledged biomarker of tissue hypoperfusion [65]. In addition, sepsis nonsurvivors had higher cytochrome c levels than survivors [64]. Eleftheriadis et al. [66] have demonstrated that plasma cytochrome c and IL-6 levels in patients after hemodialysis are significantly increased and positively correlated. Because cytochrome c is located in the mitochondrial membrane gap, it also can serve as an effective biological marker of mitochondrial damage. However, the quality and quantity of clinical studies on cytochrome c in sepsis are insufficient; therefore, the clinical importance of cytochrome c remains to be further studied.

## 5. Succinate

Succinate is an important metabolite in the tricarboxylic acid cycle. Succinate is both an important participant in energy metabolism and an mtDAMP, playing an important role in the inflammatory response. Succinate can bind to G protein-coupled receptor (GPR) 91 to achieve signal transduction. GPR91 is highly expressed on the surface of dendritic cells and plays an important role in diseases of adipose tissue, liver, immune system, retina, and kidney, but its signal cascade is not clear [67]. He et al. [68] have demonstrated that succinate can stimulate GPR91 and lead to accumulation of inositol triphosphate, calcium mobilization, and extracellular signal regulation kinase (ERK) phosphorylation. Succinate induces the migration of dendritic cells and cooperates with TLR ligands to produce proinflammatory cytokines. In addition, succinate enhances dendritic cell-mediated T cell activation, thereby enhancing the cellular immune response [67]. Tannahill et al. [69] have demonstrated that after the release of succinate from mitochondria into the cytoplasm, succinate can stabilize the hypoxia-inducible factor 1*α*, which directly leads to an increase of the transcription level of IL-1*β* mRNA. Upon LPS stimulation, the major metabolism of bone marrow-derived macrophages can be transformed from oxidative phosphorylation to glycolysis (Warburg effect), which increases the accumulation of succinate and is detrimental to the outcome of sepsis. In fact, some scholars believe that the reverse Warburg effect may improve the prognosis of sepsis [70]. Bakalov et al. [71] used nuclear magnetic resonance to explore metabolomics in a new survival model of sepsis in *Drosophila melanogaster* to determine the metabolic spectrum. Sepsis survivors had a metabolic signature characterized by decreased succinate. Lactic acid is one of the important products of glycolysis, so high plasma lactic acid level is considered to be an important indicator of poor prognosis of sepsis. Piel et al. [72] have identified a novel cell-permeable succinate prodrug (NV118) that can attenuate lactate production. NV118 releases succinate that enters the Krebs cycle to enable ATP production via oxidative phosphorylation [73]. Protti et al. [74] have demonstrated that ex vivo mitochondrial oxygen consumption is improved by succinate in skeletal muscle taken from septic rats. Energy metabolism and immune regulation of the body are important factors to determine the outcome of sepsis. The long-term substitution of glycolysis for oxidative phosphorylation is one of the signs of poor prognosis of sepsis. Succinate is involved in both physiological processes, so it is important to clarify the role of succinate in sepsis. Chapela et al. [75] have demonstrated that parenteral succinate can reduce production of systemic ROS in septic rats, which can potentially damage cells and destroy tissue. However, the level of creatinine, which reflects renal functions, was not reduced with the administration of succinate in this study. The correlation between systemic ROS levels and creatinine was not identified. The study of succinate may open a new way for the treatment of sepsis in the future. More robust clinical trials are needed in the future to identify the role of succinate in sepsis.

## 6. Cardiolipin (CL)

CL is a phospholipid dimer formed by glycerol that connects two phosphatidyl residues on the mitochondrial inner membrane. At present, pulmonary infection is still the most common source of sepsis. Therefore, it is important to reveal the mechanism of lung injury in infection for the prevention and treatment of sepsis. Ray et al. [76] found high levels of CL in the lung fluid of patients with bacterial pneumonia and mice. They further demonstrated that CL antagonized surfactant functions, resulting in high-tension pulmonary edema on the lung surface. In addition, CL can also reduce the activity of alveolar epithelial cells. Atp8b1 is a P-type ATPase transmembrane lipid pump. Atp8b1 bound and internalized CL from extracellular fluid via a basic-residue-enriched motif. Administration of a peptide encompassing the cardiolipin-binding motif or Atp8b1 gene transfer in mice lessened bacteria-induced lung injury and improved survival. Thus, excess CL in pulmonary fluid has a harmful role in bacterial pneumonia, and Atp8b1 can reduce tissue damage in inflammation by transferring CL into cells. Iyer et al. [77] identified a NLRP3 agonist that can activate NLRP3 without mass production of ROS. They showed that mitochondrial CL binds directly to NLRP3 and activates the NLRP3 inflammasome. Dieude et al. [78] have found that CL can stimulate *γδ*T cells in the spleen and liver of healthy mice to rapidly secrete cytokines such as IFN-*γ* and RANTES. CD1d is considered to be an effective recognition receptor of CL, and blocking the binding of CL to CD1d and preventing damage of the lung, liver, and spleen may be an effective means to prevent sepsis from developing into MODS.

## 7. N-Formyl Peptides (NFPs)

NFPs are the earliest known leukocyte chemical peptides. Zhang et al. [79] have demonstrated that NFPs play a crucial role in SIRS. By activating formyl peptide receptor- (FPR-) 1, NFP promotes calcium influx and phosphorylation of MAPK in polymorphonuclear neutrophils (PMNs), thereby activating human PMNs to release matrix metalloproteinase-8 and IL-8 (a potent chemokine). Acute respiratory distress syndrome (ARDS) is a pulmonary disease characterized by intractable hypoxemia. It is one of the most common complications of sepsis and one of the most difficult problems in the treatment of sepsis. Dorward et al. [80] have revealed that the mitochondrial NFP-FPR1 signal is a key driver of aseptic acute lung injury and potential therapeutic target for ARDS. By activating FPR1, which is expressed in alveolar epithelial cells, NFPs activate neutrophils and then cause them to migrate into the lung, leading to severe lung injury. This lung injury is significantly relieved when FPR1 is pharmacologically blocked. Wenceslau et al. [81] have found in a rat model of hemorrhagic shock that NFPs can cause lung injury by increasing the expression of neutrophil elastase in the lungs, and inducible nitric oxide synthase and cell division control protein 42 in the airways. They have also observed that contraction of the trachea and bronchi caused by NFPs is concentration-dependent. Martinez-Quinones et al. hypothesized that increased frequency of peritoneal cavity lavage may reduce the risk of SIRS via decreasing the levels of mtDAMPs according to their pilot study [21]. Based on Martinez's finding, we suggest that more studies are needed to determine the role of extracellular NFPs in SIRS and their possible clinical applications.

## 8. ATP

Mitochondria are the main sites of oxidative phosphorylation, which is the main process of ATP production [82]. Extracellular ATP (eATP) induces a series of immune responses and participates in the regulation of a variety of cellular functions [83]. Burnstock and Kennedy [84] named ATP-recognizing receptors as purinoceptor. Among them, the type P2 receptor has received extensive attention and is divided into P2X and P2Y according to the nature of its receptor (P2Y GPRs and P2X ligand-gated ion channels). eATP can activate multiple pathways such as phospholipase A2, phospholipase D, MAPK, and NF-*κ*B, induce the production and release of mature IL-1*β*, IL-6, and TNF-*α*, and participate in the pathogenesis of inflammatory diseases. Activation of calcium-dependent phosphatase/T cell activation of nuclear factor of activated T cells leads to the synthesis of proinflammatory factors such as cyclooxygenase-2 and inducible nitric oxide synthase [85]. In addition, ATP is recognized by P2Y2 receptors on monocytes and induces its recruitment to apoptosis sites [86]. Cauwels et al. [87] have demonstrated that systematic clearance of eATP by apyrase prevents accumulation of IL-1*β* and production of TNF-*α*, IL-10, and other cytokines. The intestinal tract is considered to be the driver of MODS in sepsis, and intestinal barrier destruction is an important factor. Our research has previously [10] shown that in a mouse sepsis model, intraperitoneal injection of P2X7R antagonist A740003 inhibits activation of the ERK/NF-*κ*B pathway of M1 macrophages in the intestinal tract, alleviates intestinal barrier damage, and reduces mortality. Arulkumaran et al. [88] have demonstrated that selective P2X7 receptor antagonist A438079 significantly improves systemic inflammatory response and renal dysfunction in sepsis. IL-1*β* levels in kidneys of A438079-treated animals are significantly lower than those of untreated animals. In addition, ATP can activate NLRP3 inflammasomes by promoting K^+^ outflow [89]. In sepsis, hepatic eATP activates P2X7 receptors, leading to sepsis-related liver injury [90]. The chemical blockade of P2X7 extensively prevents tissue damage, apoptosis, cytokine production, and activation of inflammatory signaling pathways in the liver caused by sepsis. CD39 (ENTPD1) scavenges eATP to produce adenosine, which limits P2X7 activation and leads to A_2A_ receptor excitation [91, 92]. Savio et al. [93] have shown that the expression of CD39 in macrophages limits the proinflammatory signal of ATP-P2X7 receptor, and CD39 gene deletion aggravates experimental liver injury caused by sepsis.

However, Ho et al. [94] have suggested that ATP-triggered inflammatory responses in sepsis are not entirely harmful. They have found that there is a factor in the plasma of sepsis patients that can inhibit activation of ATP-dependent inflammatory pathways, thereby significantly inhibiting production of IL-1*β* in THP-1 cells, leading to immune paralysis. Csoka et al. [95] have shown that activation of ATP-dependent P2X4 receptors in macrophages helps to kill bacteria and avoids organ damage caused by sepsis. The allosteric activator of P2X4 receptor ivermectin can prevent the spread and death of bacteria in sepsis. Is ATP an angel or a devil in sepsis? This is the problem that not only ATP but also all mtDAMPs have to face. Therefore, the study of mtDAMPs cannot be limited to one effect caused by one receptor or one signaling pathway. It is necessary to evaluate the comprehensive effect of mtDAMPs *in vivo*.

## 9. Mitochondrial Transcription Factor (TFAM)

TFAM is closely bound to mtDNA under physiological conditions, which helps to stabilize the normal structure of mtDNA. When the mitochondria are damaged, TFAM and mtDNA are released into the cytoplasm together [96]. Julian et al. [97] have demonstrated that TFAM promotes the TNF-*α* release via RAGE- and TLR9-responsive plasmacytoid dendritic cells in the spleen. Chaung et al. [98] have demonstrated that the plasma TFAM level is significantly increased in patients with hemorrhagic shock, and TFAM could promote the release of inflammatory factors such as IL-6 and TNF-*α*. West et al. [99] have suggested that TFAM has an important role in maintaining the stability of mtDNA. When TFAM is deficient, the stability of mtDNA is disrupted, and it escapes into the cytoplasm, where the mtDNA activates the cGAS/STING/IFN-I signaling pathway. In summary, TFAM, as an important mtDAMP, plays a dual role in the activation of signaling pathways: on the one hand, TFAM can prevent the release of mtDNA into the cytoplasm and activate cGAS and other nucleic acid receptors; on the other hand, TFAM itself, as a DAMP, can amplify the inflammatory response and cause damage to important organs. Therefore, the synergistic effect of TFAM and mtDNA should be further studied. The independent effect of TFAM in sepsis also should be further clarified.

## 10. Conclusion

mtDAMPs play an important role in the development of sepsis. On the one hand, cytokine-induced inflammatory response protects the body against PAMPs. On the other hand, excessive inflammatory reactions (such as SIRS) and disorders of the immune system (such as immune paralysis) can seriously damage the organ function of the body, leading to the occurrence, aggravation, and even progression of sepsis to MODS and death. In addition, mtDAMP-mediated signaling pathway activation can further damage mitochondria and leads to more mtDAMP release, which is a vicious circle. Interrupting this cycle by the antagonism of these mtDAMP-related receptors and inflammasomes is hopefully an important target in the prevention and treatment of sepsis.
